# Comparing the cobas Influenza A/B Nucleic acid test for use on the cobas Liat System (Liat) with rapid antigen tests for clinical management of Japanese patients at the point of care

**DOI:** 10.1371/journal.pone.0276099

**Published:** 2022-10-27

**Authors:** Hiroshige Mikamo, Yusuke Koizumi, Yuka Yamagishi, Nobuhiro Asai, Yuko Miyazono, Toshikazu Shinbo, Michiko Horie, Kenichi Togashi, Elissa M. Robbins, Nobuo Hirotsu

**Affiliations:** 1 Aichi Medical University, Nagakute, Aichi, Japan; 2 Wakayama Medical University, Wakayama, Wakayama, Japan; 3 Kochi University, Nankoku, Kochi, Japan; 4 Miyazono Naika Clinic, Nerima, Tokyo, Japan; 5 Shinbo Child Clinic, Yokohama, Kanagawa, Japan; 6 Roche Diagnostics KK, Minato, Tokyo, Japan; 7 Roche Diagnostics, Pleasanton, California, United States of America; 8 Hirotsu Medical Clinic, Kawasaki, Kanagawa, Japan; Children’s National Hospital, George Washington University, UNITED STATES

## Abstract

**Background:**

Rapid diagnosis of influenza is critical in preventing the spread of infection and ensuring patients quickly receive antiviral medication to reduce the severity and duration of influenza symptoms, whilst controlling the spread of the causative virus. In Japan patients are often administered anti-influenza medication following a positive rapid antigen detection test (RADT) result. However, the sensitivity of RADTs can lead to false negative results. The cobas^®^ Influenza A/B Nucleic acid test for use on the cobas Liat^®^ System (Liat) is a molecular point-of-care method that can provide a more sensitive alternative to RADTs for rapid influenza diagnosis and treatment.

**Methods:**

In this prospective multicenter study, diagnostic performance of the Liat test was compared with RADTs in patients presenting with influenza-like-illness. Test performance was also assessed by time since symptom onset.

**Results:**

Of 419 patients enrolled, 413 were evaluable for all designated tests. Most patients had type-A infection, and only one patient had influenza type B. In 413 patients, the sensitivity and specificity (95% CI) of the Liat test were 99.5% (97.2–99.9%) and 99.5% (97.4–99.9%), respectively, and were 79.7% (73.5–84.7%) and 95.4% (91.7–97.5%) for RADTs. For patients tested <12 hours from symptom onset, the Liat test had significantly higher sensitivity than RADTs (p<0.0001).

**Conclusion:**

Overall, compared with standard of care RADTs, the Liat test was more sensitive and specific in children and adults, particularly in the early stages of infection. Greater sensitivity can enable earlier diagnosis and may better inform appropriate antiviral treatment decisions.

## 1. Introduction

Estimates suggest that influenza causes 3–5 million infections each year leading to severe illness and up to 650,000 respiratory deaths [1]. Historically, in Japan, the consequences of influenza have led to high rates of mortality and associated encephalopathy [2–4]. As such, influenza-related hospitalization, mortality and encephalopathy are reportable to the National Epidemiological Surveillance of Infectious Diseases [5–7].

Rapid antigen detection tests (RADTs) are commonly used as part of influenza diagnosis in Japan, but the reported lower sensitivity of RADTs may result in false-negative diagnoses, especially in early phases of influenza infection when viral load is lower [8–12]. In order to reduce the duration and severity of influenza symptoms, whilst controlling the onward spread of the causative virus, it is common for patients to receive treatment with antiviral drugs within 48 hours of symptom onset [5, 13, 14]. Approximately 7–8 million patients are treated each year with anti-influenza drugs in Japan, 80–95% of which were administered within 48 hours of symptom onset [7]. This is in line with Japanese guidelines for the treatment of influenza [15], and may contribute to lower influenza-related mortality versus other countries [7, 16]. In Japan, outpatients are generally prescribed with anti-influenza mediation if they test positive for influenza by RADT [7]. Use of RADTs for the confirmation of influenza infection is reimbursed by the Japanese health insurance system, therefore they are widely used [17]. Furthermore, the guidelines for anti-influenza medication use from the Japanese Society for Infectious Diseases note that even with a negative test, or without a test result, patients may receive anti-influenza medication if the clinician suspects influenza [18, 19]. In other countries anti-influenza medication is usually reserved for at-risk patients e.g. young children or the elderly; however, effective use can help to prevent secondary transmission of symptomatic influenza, and future outbreaks [7, 20].

The cobas^®^ Influenza A/B Nucleic acid test for use on the cobas Liat^®^ System (Liat) is an FDA-cleared molecular point-of-care (POC) test which uses real-time reverse transcription polymerase chain reaction (RT-PCR) to detect influenza A and influenza B virus RNA in ~20 minutes [21, 22]; this test has not been reviewed or approved by MHLW in Japan. Molecular methods such as RT-PCR are standard for routine laboratory confirmation of influenza due to their high sensitivity and specificity and faster turnaround times than viral culture [23]. There is evidence that POC tests for influenza, including antigen tests, can influence clinical decision-making, and lead to fewer blood tests and chest X-rays [24]. Molecular testing in particular may be able to detect viral RNA from nasopharyngeal specimens over longer time periods, allowing greater sensitivity and more accurate diagnosis [18].

With the advent of the global coronavirus (COVID-19) pandemic, there is an urgent need to rapidly and accurately diagnose causative pathogens of influenza-like illness (ILI) due to the overlapping signs and symptoms of influenza A/B and SARS-CoV-2 infection [25]. The aim of this study was to assess the clinical performance of molecular POC testing using the Liat test versus the standard of care testing using RADTs in patients presenting with ILI during flu season in Japan.

## 2. Materials and methods

### 2.1 Study design and ethics

This was a prospective multicenter, observational study comparing the performance of the Liat influenza A/B test (Roche Diagnostics GmbH, Mannheim, Germany) [22] with the standard of care, RADTs, in patients with ILI and carried out between December 2018 and March 2019. Ethical approval for this study was obtained from the Ethics Review board at each participating center: Aichi Medical University, Nagakute, Aichi (IRB: 3021); Miyazono Naika Clinic, Tokyo; Shinbo Child Clinic, Yokohama, Kanagawa; and Hirotsu Medical Clinic, Kawasaki (all approved by Shiba-palace clinic IRB: 141941_rn-25550). This study was conducted in compliance with the Declaration of Helsinki (2013 revised Forta Reza) and the Ethical Guidelines for Medical Research Involving Human Subjects (partially revised on February 28, 2017). All patients included in the study were required to provide written informed consent either themselves, or via a legally acceptable representative before sample collection.

### 2.2 Patients

Patients presenting with ILI at a hospital and three clinics in Japan were included in the study if they visited the hospital or clinic within 48 hours of the onset symptoms associated with ILI. ILI was defined as pyrexia of 38°C or higher or body temperature increase of 1°C or more, with at least one acute respiratory or systemic symptom (headache, chills, pain in muscles or joints, fatigue, cough, sore throat, or stuffy nose). Patients were excluded from the study if: they had used anti-influenza drugs, traditional Chinese medicines or complementary therapies for influenza within the week before their hospital/clinic visit; had participated in the study within the week before the visit; or the physician judged their inclusion was inappropriate.

### 2.3 Study procedures

At the patient’s first visit following inclusion into the study, their clinical information (sex, age, temperature, and influenza vaccination status), the date and time of consultation, the onset of symptoms, any respiratory or systemic symptoms and any medication prescribed were recorded. Nasopharyngeal swabs were also obtained from each subject for routine clinical use, and were taken from the opposite side of the nostril immediately after collection of influenza specimens. Swab samples were immediately suspended in universal transport medium (UTM) and 200 μL of the UTM suspension was then added to assay tubes for testing with the Liat test, according to the manufacturer’s instructions. If samples were not immediately tested with the Liat test, they were stored at 2–8°C for testing on the same day.

At the first visit, patients were given a form, on which they recorded their symptoms for 1 week. Completed forms were returned to each clinical site by mail. One week after the initial consultation, symptom duration, physical examination for symptoms, date and time of follow-up, and any other influenza medications prescribed were noted. The physicians made their final diagnosis based on information collected from the patient.

### 2.4 Testing

Samples were tested using an RADT, following manufacturers’ instructions as per the standard operating procedure of each study center. RADTs included were: Quick Chaser Flu A,B (Mizuho Medy Co. Ltd., Saga, Japan), BD Veritor^TM^ System Flu A+B (Becton, Dickinson and Company, Sparks, Maryland, USA), BRIGHTPOC^®^ FluNeo (Shionogi, Osaka, Japan), Quick Navi^TM^-Flu/Quick Navi^TM^-Flu2 (Otsuka, Chiyoda, Japan), and ImunoAce Flu (Tauns Laboratories, Inc. Shizuoka, Japan). Samples were also tested with the Liat test and results were verified by a second person at each study center. Residual samples were stored frozen at ≤-70°C in case additional testing was later required.

Samples with discordant results between the Liat test and RADTs were additionally tested using the RealStar^®^ Influenza RT-PCR Kit 2.0 (Altona Diagnostics).

### 2.5 Data analysis and interpretation of results

Overall agreement between Liat test and RADTs was evaluated. Concordant samples that were both positive or both negative between the Liat test and RADTs were not further tested. Discordant results between Liat test and RADTs were then adjudicated with the RealStar assay, and the majority result was used to define the final result status.

Clinical performance was evaluated using the RealStar-adjudicated final results as the comparator to calculate sensitivity and specificity of the Liat test and RADTs with their respective 95% confidence intervals (CIs). P-values comparing sensitivity and specificity were calculated using the 2-sample chi-square test for equal sensitivities, with continuity correction. Sensitivity and specificity of the test assays were also analyzed by time from symptom onset.

Data analyses were performed using either JMP 13.2.1 (SAS Institute Inc.) or SAS/STAT^®^ software and in accordance with the FDA guidelines [26].

## 3. Results

### 3.1 Patients

**Fig 1:**
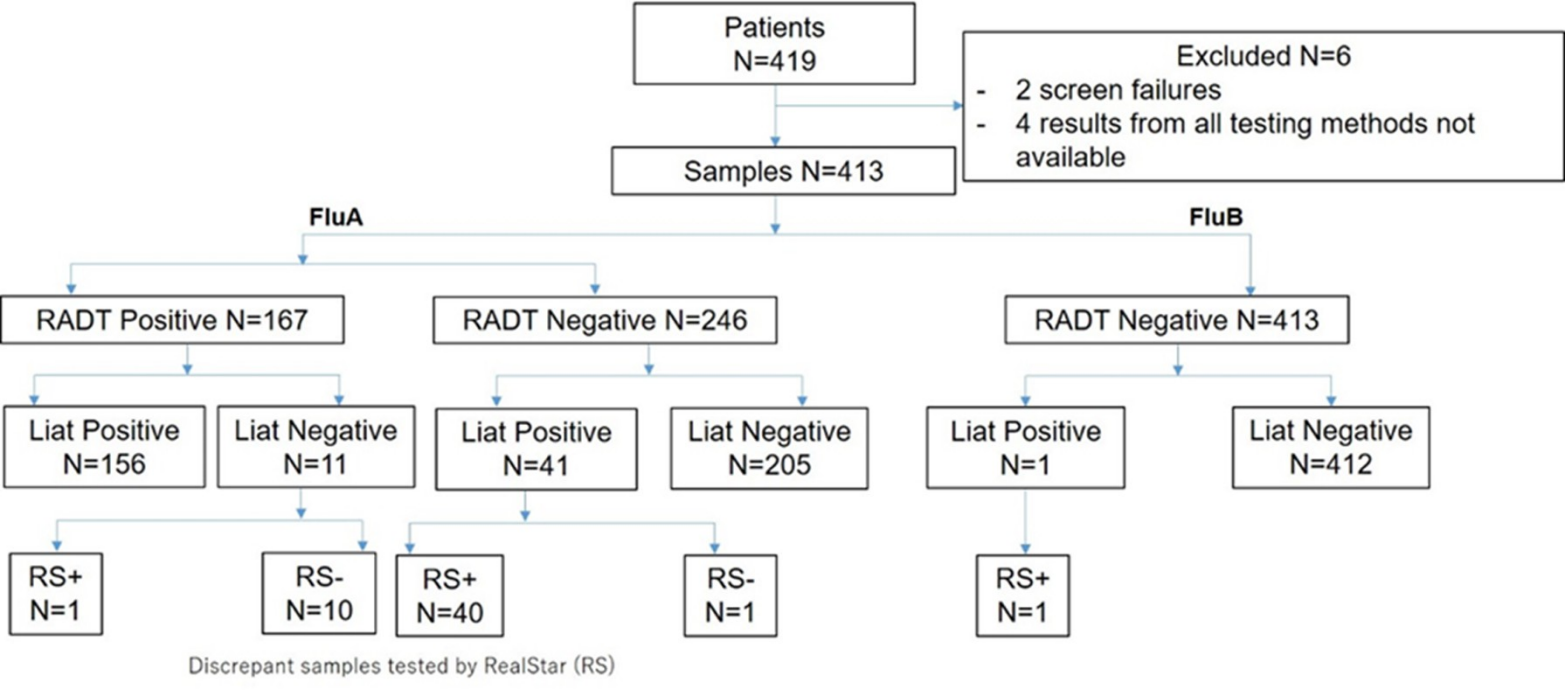
Patient flow. Discrepant samples were tested by RealStar; invalid RS samples (N = 4) were excluded from further analysis. RADT, rapid antigen diagnostic test; RS, RealStar.

A total of 419 patients were enrolled, of which 413 patients were evaluable for all necessary tests within the study (Fig 1). Median patient age was 13 years (range 0–86 years) and the median time to visit at the clinic or hospital was 8 hours from onset of ILI symptoms (Table 1). Most patients had type-A infection, and only one patient had influenza type B. Approximately 45% (186/413) of patients had received seasonal vaccination for influenza A/B. Additional patient characteristics are summarized in Table 1. Data are not reported for initial physical examination and 1-week follow-up.

**Table 1:** Patient characteristics.

Characteristic	Total (N = 413)	
**Age, median**	13 years	[5–37]
**≤5 years**	110	(26.6%)
**6–15 years**	107	(25.9%)
**16–59 years**	175	(42.4%)
**≥60 years**	21	(5.1%)
**Female**	209	(50.6%)
**Vaccination** ^**a**^	186	(45.0%)
**Time from symptom onset, median**	8 hours	[4–17]
**Body temperature, median**	38.0°C	[37.3–38.6]
**Headache**	190	(46.0%)
**Chills**	238	(57.6%)
**Pain in muscles or joints**	139	(33.7%)
**Fatigue**	190	(46.0%)
**Cough**	215	(52.1%)
**Sore throat**	145	(35.1%)
**Stuffy nose**	159	(38.5%)

Data are means (%) or medians [Interquartile range, IQR].

^a^Includes vaccinations administered in the same season.

### 3.2 Agreement between RADTs and the Liat test

Of the 413 samples tested by RADT, 143 (34.6%) were tested using Quick Chaser Flu A,B; 61 (14.8%) using Quick Navi-Flu2; 60 (14.5%) using ImunoAce Flu; 56 (13.6%) using Quick Navi-Flu; 54 (13.1%) using BD Veritor System Flu A+B; and 39 (9.4%) using BRIGHTPOC FluNeo. In total, 156 samples were both RADT and Liat test influenza A positive. Of the 42 samples that were Liat test influenza A positive and RADT influenza A negative, 40 were confirmed positive by RealStar, one was negative by RealStar, and one was excluded from analysis for an invalid RealStar result. Of the 14 samples that were RADT influenza A positive and Liat test influenza A negative, 10 were confirmed as negative, one was positive by RealStar, and three were excluded from analysis for invalid RealStar results (Fig 1). For influenza B, one sample was positive using the Liat test, and negative with the RADTs. This sample was confirmed influenza B positive with the RealStar assay; during discrepancy testing, this sample was also confirmed influenza A positive using both RADTs and the Liat test, but influenza A negative with the RealStar assay (S1 Table). The Liat test was 99.5% (196/197) sensitive (95% CI 97.2–99.9%) and 99.5% (215/216) specific (95% CI 97.4–99.9%) for the detection of influenza A (Table 2). The Liat test was significantly more sensitive and specific than the RADTs overall sensitivity of 79.7% (95% CI 73.5%–84.7%) and specificity of 95.4% (95% CI 91.7–97.5%) for influenza A (Table 2). The specificity for influenza B was 100% (95% CI 99.1–100%) for both the Liat test and RADT, however, the sample size (n = 1) was too small for sensitivity calculations.

**Table 2:** Clinical performance of the Liat test compared with RADTs for influenza A detection.

	Liat influenza A			RADTs influenza A		
Final result	Positive	Negative	Total	Positive	Negative	Total
Positive	196	1	197	157	40	197
Negative	1	215	216	10	206	216
Total	197	216	413	167	246	413
Sensitivity (%, 95% CI)	99.5% (97.2%–99.9%)*			79.7% (73.5%–84.7%)*		
Specificity (%, 95% CI)	99.5% (97.4–99.9%)^†^			95.4% (91.7–97.5%)^†^		

Note invalid RealStar results were excluded from these calculations.

*The p-value from 2-sample chi-square test for equal sensitivities with continuity correction is <0.0001.

†The p-value from 2-sample chi-square test for equal specificities with continuity correction is 0.015.

CI, confidence interval; RADT, rapid antigen diagnostic test; RS, RealStar.

The RADT tests were also more sensitive in younger age groups in this study; sensitivity ranged from 87.5% (95% CI 73.2–95.8%) in children aged under 6 years, decreasing to 53.3% (95% CI 26.6–78.7%) in the over 60 age group. In comparison, the sensitivity of the Liat test ranged from 92.5% (95% CI 79.6–98.4%) in those aged under 6 years to 80.0% (95% CI 51.9–95.7%) in the over 60 age group.

### 3.3 Liat test performance versus symptom onset

In patients with their hospital/clinic visit <12 hours from symptom onset, the Liat test demonstrated significantly higher sensitivity (p<0.0001, 2-sample chi-square test for equal sensitivities with continuity correction). The Liat test detected 100% (127/127) of influenza-positive patients compared with 75.6% (96/127) for the RADTs, which generated 31 false negative results (Table 3). The Liat test demonstrated consistently higher sensitivity and specificity than RADTs across all three time periods (<12 hour, 12–24 hour and 24–48-hours).

**Table 3:** Comparison of the assays’ performance against final results.

	Assay	<12 hours	12–24 hours	24–48 hours
**Sensitivity** (% [n/N; 95% CI])	**Liat**	100.0% (127/127; 97.1%–100%)*	97.9% (46/47; 88.9%–99.6%)	100.0% (23/23; 85.7%–100%)
	**RADT**	75.6% (96/127; 67.4%–82.2%)*	89.4% (42/47; 77.4%–95.4%)	82.6% (19/23; 62.9%–93.0%)
**Specificity** (% [n/N; 95% CI])	**Liat**	99.2% (124/125; 95.6%–99.9%)	100.0% (73/73; 95.0%–100%)	100.0% (18/18; 82.4%–100%)
	**RADT**	96.8% (121/125; 92.1%–98.8%)	94.5% (69/73; 86.7%–97.9%)	88.9% (16/18; 67.2%–96.9%)

*p<0.0001 by 2-sample chi-square test for equal sensitivities with continuity correction for “<12 hours” time period. Although for all other comparisons the sensitivity and specificity by Liat test are consistently higher, the difference in sensitivity or specificity is not statistically significant due to small sample size.

### 3.4 Treatment for influenza

RADTs are routinely performed in patients presenting with symptoms, but the results are not required for antiviral agent administration. Anti-influenza treatments were prescribed to >50% of patients (207/413) within the study (Table 4). The most commonly prescribed antiviral was baloxavir marboxil, prescribed to 78.3% (162/207) of all patients with both a valid Liat test and RADT result. The median age of patients receiving baloxavir marboxil was 16 years. The second most commonly prescribed antiviral agent was osteltamivir; prescribed to 12.1% (25/207) of patients with a median age of 2 years. Over 90% of prescriptions for osteltamivir were for children younger than 8 years old.

**Table 4:** Antiviral influenza medications administered for all patients with both a valid Liat test and RADT result.

Antiviral Treatment	Subjects	
	N	%
**Baloxavir marboxil**	162	78.3
**Oseltamivir**	25	12.1
**Zanamivir**	13	6.3
**Peramivir**	5	2.4
**Laninamivir**	2	1.0
**Total**	207	100.0

RADT, rapid antigen diagnostic test.

## 4. Discussion

This study found that missed diagnosis of influenza is more likely when using RADTs compared with the Liat influenza A/B test, as RADTs were less sensitive and specific compared with the Liat test. These findings are consistent with previously published studies of RADTs, which found the tests are adequately specific, but have a poor sensitivity that can vary depending on the testing population [8–10, 12, 27, 28].

In this study, the majority of patients visiting the hospital or clinic underwent testing within 12 hours of symptom onset. For these patients, the Liat influenza A/B test was significantly more sensitive than RADTs when detecting influenza A. Beyond 12 hours from symptom onset, the Liat test was consistently more sensitive than RADTs, but these differences were not significant due to the small sample size. Previous research has demonstrated that RADT sensitivity is lower within 24 hours of symptom onset and higher between days 2–4 [29, 30]. Scheduled visits in this study were earlier in disease course than in other studies, which may have contributed to the lower sensitivity of RADTs observed. However, early assessment and diagnostic testing is reflective of ILI-management practices in Japan [31]. Further, although previous research has demonstrated high diagnostic performance of the Liat test, reported sensitivities and specificities do not always reach 100% [32, 33].

Another possible reason for discrepancies between results from RADTs and the Liat influenza A/B test could be due to the visual interpretation required for some RADTs, which can allow for error or misinterpretation [34, 35]. However, RADTs with digital readouts were also included in this study. Alternately, higher rates of false results from RADTs may be due to their operational workflow and open design that typically requires multiple handling steps and the addition of sample reagents to an open testing area, thus increasing the risk of contamination. The cobas Liat System is a fully closed system which reduces the risk of cross-contamination while preserving sample integrity [34].

To reduce the duration and severity of influenza symptoms, and help prevent secondary transmission of the virus, antiviral medication should be administered within 48 hours of symptom onset [5, 13, 36]. On the other hand, inappropriate/over-prescription of antiviral medication can increase the risk of antiviral resistance [37]. Previous studies found that triaging patients with suspected influenza using RADTs led to earlier treatment with antiviral medication [9], and this effect may be expected to increase when using a more sensitive molecular POC such as the Liat test.

In this study, the most commonly prescribed antiviral medication was baloxavir marboxil, potentially because unlike earlier drugs, this most recently approved antiviral has the advantage of a single-dose regimen [38]. A test-and-treat strategy using a more accurate PCR-based test could increase the appropriate and judicious prescription of anti-influenza medication, and may also reduce unnecessary antibiotic use that can increase the risk of antibiotic resistance and lead to common side effects [20, 39–45]. Therefore, detecting significantly more newly infected influenza patients can potentially offer greater clinical benefits for patient management and outcomes.

Since this study was conducted during the 2018–2019 influenza season prior to the emergence of SARS-CoV-2, the global need for high sensitivity respiratory tests has become even more critical now in the context of the COVID-19 pandemic. With the current scarcity of rapid molecular tests approved for diagnosis of SARS-CoV-2 infection, point-of-care testing for influenza A/B may be used by clinicians during upcoming seasons to quickly rule in or rule out influenza in patients presenting with similar respiratory symptoms. The Liat Influenza A/B test was discontinued by Roche in 2021 in response to ongoing pandemic testing needs and is replaced by Liat tests for Influenza A/B & RSV (respiratory syncytial virus) and SARS-CoV-2 & Influenza A/B, which have demonstrated similarly high performance for detecting influenza as the Liat Influenza A/B test [22, 46–48]. The selection of highly accurate molecular POC tests for influenza A/B can aid in the differentiation and diagnosis of this disease from COVID-19, potentially reducing the need for additional tests and improving infection control by correctly identifying influenza cases for appropriate patient triage and treatment decisions.

One limitation of this study is that only the RealStar assay was used to adjudicate discrepancies between results from the Liat test and RADTs. The findings from this study may have been strengthened with additional confirmatory testing. Only testing samples with discrepant results may have limited the generalizability of this study. In addition, only one patient with influenza B was included in the study, which does not represent the expected pattern of infection. Though the participating sites were limited to Aichi, Kanagawa, and Tokyo, there were no local differences in prevalence in Japan during the 2018–2019 influenza season [49, 50] and, therefore, the study sample can be considered representative of the larger population.

## 5. Conclusions

Compared with RADTs that are commonly used in clinical practice in Japan, the Liat influenza A/B test has significantly greater sensitivity for the diagnosis of influenza in children and adults, particularly in the early stages of infection when antiviral treatment is most effective. This greater sensitivity can enable earlier diagnosis and delivery of treatment, and lead to potential improvements in clinical outcomes and effective infection control.

## Supporting information

S1 Table Discrepancy analysis for influenza A/B test results, N = 53. (DOCX)
