# Supplementary material for: Comparing the cobas Influenza A/B Nucleic acid test for use on the cobas Liat System (Liat) with rapid antigen tests for clinical management of Japanese patients at the point of care
Source: PLoS One. 2022 Oct 27;17(10):e0276099. doi: 10.1371/journal.pone.0276099 (PMC9612487; doi:10.1371/journal.pone.0276099)
Supplement: S1 Table — (DOCX) [file pone.0276099.s001.docx]

**Supplementary Table 1. Discrepancy analysis for influenza A/B test results, N=53**

| **Final Result** | **Type** | **RADT** | **Liat** | **RealStar** | **Frequency** |
| --- | --- | --- | --- | --- | --- |
| **Positive** | A | - | + | + | 40 |
|  | A | + | - | + | 1 |
|  | B | - | + | + | 1* |
| **Negative** | A | - | + | - | 1 |
|  | A | + | - | - | 10 |
| **Total** | | | | | 53 |

*This sample was also RADT+ / Liat^®^ + / RS- for Flu A, there were no other discrepant influenza B results

RADT, rapid antigen diagnostic test; RS, RealStar
